# Regulation of translation by one-carbon metabolism in bacteria and eukaryotic organelles

**DOI:** 10.1074/jbc.REV120.011985

**Published:** 2020-11-21

**Authors:** Sunil Shetty, Umesh Varshney

**Affiliations:** 1 Biozentrum, University of Basel, Basel, Switzerland; 2 Department of Microbiology and Cell Biology, Indian Institute of Science, Bangalore, India; 3 Jawaharlal Nehru Centre for Advanced Scientific Studies, Jakkur, Bangalore, India

**Keywords:** folate metabolism, RNA modifications, mitochondria, formylation, ribosome heterogeneity, MAP, methionine aminopeptidases, OCM, one-carbon metabolism, OXPHOS, oxidative phosphorylation system, PTC, peptidyl transferase center, RRF, ribosome recycling factor, SAM, S-adenosine methionine, SCP, sulfachloropyridazine, SMX, sulfamethoxazole, TCA, tricarboxylic acid, TMP, trimethoprim

## Abstract

Protein synthesis is an energetically costly cellular activity. It is therefore important that the process of mRNA translation remains in excellent synchrony with cellular metabolism and its energy reserves. Unregulated translation could lead to the production of incomplete, mistranslated, or misfolded proteins, squandering the energy needed for cellular sustenance and causing cytotoxicity. One-carbon metabolism (OCM), an integral part of cellular intermediary metabolism, produces a number of one-carbon unit intermediates (formyl, methylene, methenyl, methyl). These OCM intermediates are required for the production of amino acids such as methionine and other biomolecules such as purines, thymidylate, and redox regulators. In this review, we discuss how OCM impacts the translation apparatus (composed of ribosome, tRNA, mRNA, and translation factors) and regulates crucial steps in protein synthesis. More specifically, we address how the OCM metabolites regulate the fidelity and rate of translation initiation in bacteria and eukaryotic organelles such as mitochondria. Modulation of the fidelity of translation initiation by OCM opens new avenues to understand alternative translation mechanisms involved in stress tolerance and drug resistance.

The capacity to modulate gene expression to enhance their fitness to survive and grow in response to changing intracellular and extracellular conditions is one of the most fascinating features of the biological systems. Translation of mRNAs is a major determinant of the changes in the cellular proteome and its downstream effects, which allow the cell to respond to the external factors such as nutrients, temperature, and pH. And, the modulation of the translational apparatus is one of the ways cells employ to rapidly change gene expression. The process of translation occurs in the nexus of both the nucleotides and amino acids, whose production is coupled with the highly conserved one-carbon metabolism (OCM).

OCM acts as a central regulator of cellular one-carbon units/groups (1, 2). The one-carbon units are essential for the synthesis of metabolites including amino acids such as methionine, as well as thymidylate and purines. The impact of OCM on protein synthesis was recognized as early as the 1970s. Similar to the inhibitors of protein synthesis, the inhibitors of OCM are also employed as antibiotics. The sulfonamides (sulfa drugs) such as sulfachloropyridazine (SCP) and sulfamethoxazole (SMX) and trimethoprim (TMP) are perhaps the most prominent examples of OCM inhibitors (3). Interestingly, treatment of *Escherichia coli* with TMP inhibited translation within 15 min (4). Supplementation of the growth media with the products of OCM such as methionine, purines, and thymidine could not rescue translation in the presence of TMP, indicating a direct contribution of OCM in translation (5, 6). Unfortunately, resistance to sulfa drugs arose rapidly (7). However, recent studies have shown that OCM inhibitors could serve as effective antibiotics when additional enzymes in the pathway are inhibited (8, 9), leading to a renewed interest in these drugs. Further, perturbation of OCM in *Salmonella typhimurium* attenuates the strain to render it a potential vaccine candidate (10). In mitochondria too, perturbation of OCM affects translation, which is crucial for the synthesis of oxidative phosphorylation system (OXPHOS) (11, 12). In addition, targeting of OCM by antifolates such as aminopterin and methotrexate has been a major intervention strategy for various cancers (2, 13).

Thus, a better understanding of the regulation of protein synthesis by OCM is crucial to develop newer antimicrobial/anticancer agents to deal with emerging drug resistance. To understand the cross talk between protein synthesis and OCM, we begin by providing brief descriptions of the two pathways and then discuss how metabolites derived from OCM regulate protein synthesis.

## Overview of protein synthesis in bacteria and eukaryotic organelles

**Figure 1:**
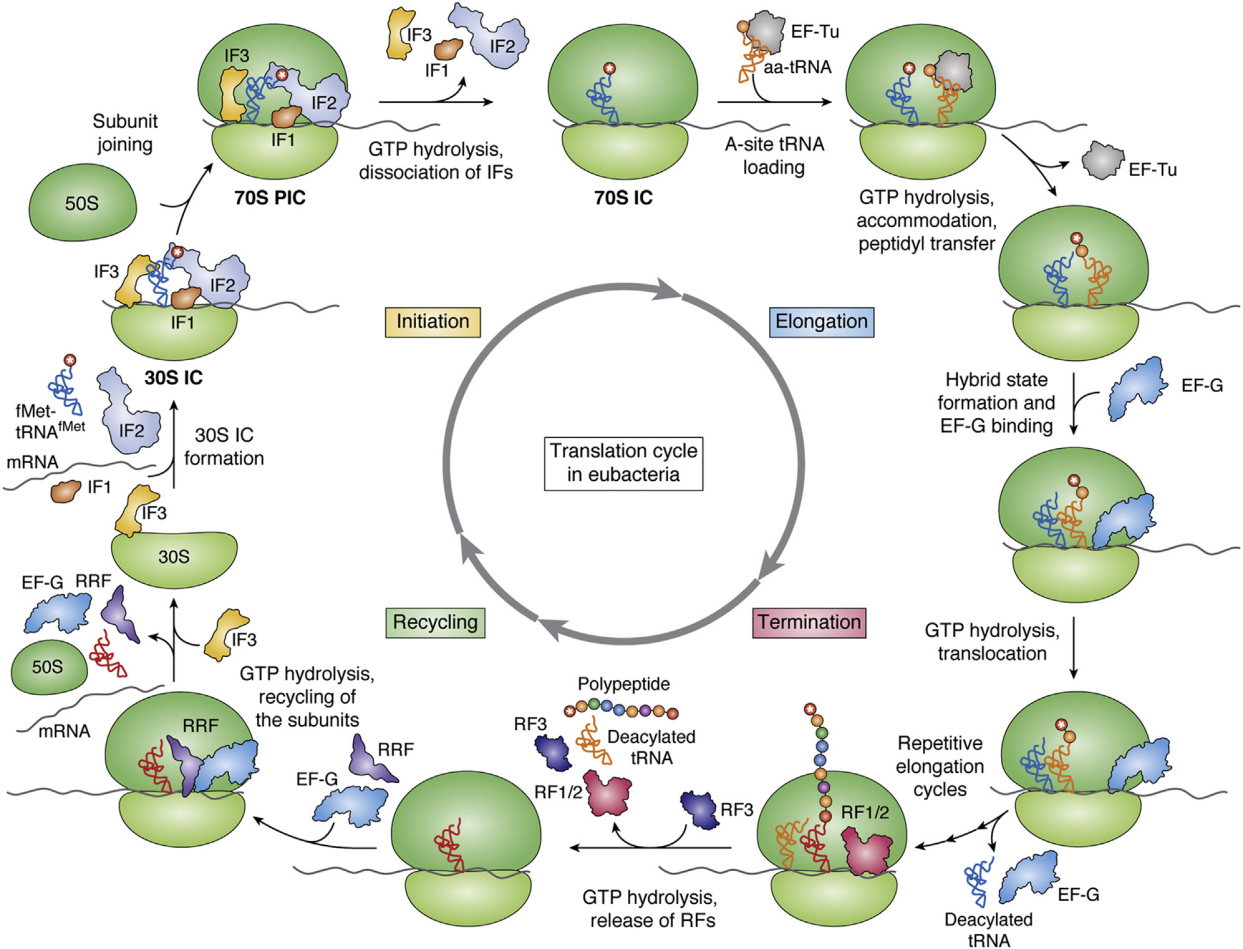
**Overview of the translation cycle in eubacteria:** The major events occurring in the translation of an mRNA are shown. The 30S IC indicates the 30S initiation complex, 70S PIC for 70S preinitiation complex, and 70S IC for 70S initiation complex. An *asterisk* within the *circle* indicates formylation of Met attached to tRNA^fMet^.

Translation of mRNA in bacteria uses 70S ribosomes made by joining of 30S (consisting of 16S rRNA and about 21 proteins) and 50S (consisting of 23S and 5S rRNAs, and about 33 proteins) subunits and occurs in four major steps: initiation, elongation, termination, and ribosome recycling (Fig. 1) (14, 15, 16). The canonical pathway of translation initiation involves the binding of mRNA with formyl-methionyl-initiator tRNA (fMet-tRNA^fMet^) to the 30S ribosomal subunit together with the initiation factors (IF1, IF2, and IF3) resulting in a 30S initiation complex (30S IC) (17, 18, 19). mRNA binding to 30S is often facilitated by pairing of the Shine–Dalgarno (SD) sequence in the mRNA (upstream of the start codon) with the anti-SD region present toward the end of the 16S rRNA (20). Subsequently, the 50S subunit joins the 30S IC to form the 70S preinitiation complex (70S PIC) *via* establishment of multiple contacts (intersubunit bridges) at the interface of the two subunits. Subunit joining leads to hydrolysis of guanosine triphosphate (GTP) bound to IF2, and the 70S PIC undergoes conformational changes with concomitant release of the initiation factors to form the 70S IC (21, 22, 23). Translation elongation follows 70S IC formation and involves elongation factor Tu (EF-Tu) mediated recruitment of a cognate aminoacyl-tRNA into the ribosomal A-site followed by peptide bond formation catalyzed by the peptidyl transferase center (PTC) in the 50S subunit. Subsequently, elongation factor G (EF-G) binds the ribosome and causes translocation of the tRNA–mRNA assembly to position the next codon in the A-site (24, 25, 26). Repeated cycles of elongation lead to synthesis of a polypeptide, which then emerges out of the peptide exit tunnel in the 50S subunit. The functions of both EF-Tu and EF-G are facilitated by GTP hydrolysis. Translation termination occurs when the ribosomal A-site encounters a stop codon (UAA, UAG, or UGA), which is recognized by release factors RF1 or RF2, leading to release of the growing polypeptide from the P-site (27). A third release factor, RF3 (which also utilizes GTP), facilitates the release of RF1 and RF2, and the resulting posttermination complex is then recycled by the ribosome recycling factor (RRF) together with EF-G and IF3 to generate ribosomal subunits for a new round of initiation (Fig. 1) (28, 29, 30). During polypeptide synthesis, incorporation of an amino acid requires at least two ATPs for the aminoacylation reaction and two GTPs for the elongation factors. As the process of translation requires enormous consumption of energy, the process is tightly coupled to the nutrient availability and the energy status of the cell.

The mitochondrial translation uses 55S ribosomes comprising a 28S small subunit (consisting of 12S rRNA and about 31 proteins) and a 39S large subunit (consisting of 16S rRNA and about 51 proteins) (31, 32, 33, 34). Even though the mitochondrial ribosomes are distinct in having an RNA to protein ratio of ∼1:2 (as opposed ∼2:1 in bacteria), the overall mechanism of protein synthesis by the two is similar (35, 36, 37, 38). However, mitochondria possess leaderless mRNAs (lacking SD sequence). Also, some of the codons in mitochondria are decoded differently (39, 40, 41). Translation initiation from such leaderless mRNAs, at least in bacteria, occurs mostly by direct recruitment of the 70S ribosomes to mRNA (42, 43, 44). Another difference in mitochondrial translation initiation is the absence of initiation factor (IF1). Instead, mitochondrial IF2 possesses an insertion, which functionally acts like IF1 (35, 45, 46). The overall mechanism of translation elongation, termination, and ribosome recycling in mitochondria occur using factors homologous to those found in bacteria, (47, 48).

## Overview of one-carbon metabolism

**Figure 2:**
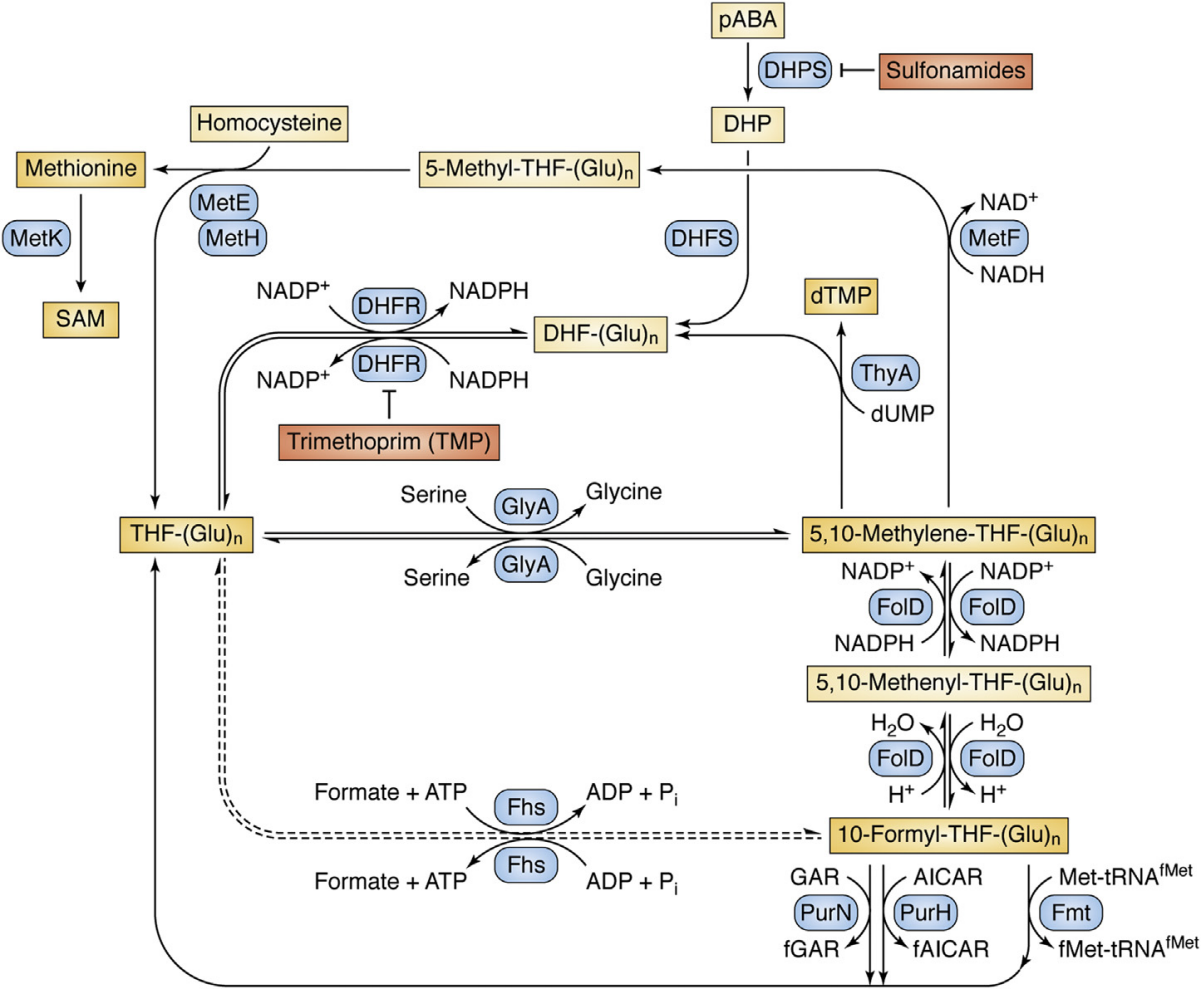
**One-carbon metabolism in bacteria:** The pathway involves dihydrofolate (DHF), tetrahydrofolate (THF), 5,10-methylene-THF, 10-formyl-THF, thymidylate (dTMP), methionine, and S-adenosylmethionine (SAM) as key products. Both DHF and THF and their derivatives possess polyglutamyl chains (n = 1–7 in *E. coli*). The Fhs reaction is shown as dashed line as it is not natural to *E. coli*. Various abbreviations are: pABA, para-amino benzoic acid; DHP, dihydropteroate; 5,10-methylene-THF, N^5^,N^10^-methylene-THF; 5,10-methenyl-THF, N^5^,N^10^-methenyl-THF; 10-formyl-THF, N^10^-formyl-THF; dUMP, deoxyuridine monophosphate; dTMP, deoxythymidine monophosphate; 5-methyl-THF, N^5^-methyl-THF; GAR, glycinamide ribonucleotide; fGAR, formylglycinamide ribonucleotide; AICAR, aminoimidazole carboxamide ribonucleotide; and fAICAR, formamidoimidazole carboxamide ribonucleotide. The enzymes are, DHPS, dihydropteroate synthase; DHFS, dihydrofolate synthase; DHFR, dihydrofolate reductase; GlyA, serine hydroxymethyltransferase; Fhs, formyltetrahydrofolate synthetase; ThyA, thymidylate synthase; MetF, 5,10-methylene-THF reductase; MetE, cobalamin-dependent homocysteine transmethylase; MetH, cobalamin-dependent methionine synthase; MetK, methionine adenosyltransferase; FolD, methylenetetrahydrofolate dehydrogenase/cyclohydrolase; Fmt, formylase; PurN, phosphoribosylglycinamide formyltransferase 1; and PurH, bifunctional AICAR transformylase/IMP cyclohydrolase.

The OCM involves various chemical transactions generating intermediates such as 5,10-methylenetetrahydrofolate, 10-formyl tetrahydrofolate, and 5-methyl tetrahydrofolate, which serve as donors of various one-carbon units (methylene, formyl, and methyl groups) for the synthesis of methionine, S-adenosine methionine (SAM), thymidylate, and purines. As these transactions of one-carbon moieties occur on the folate species (tetrahydrofolate), the pathway is also known as folate cycle/metabolism. As shown in Figure 2, OCM integrates several reactions that use tetrahydrofolate (THF) as a carrier of the one-carbon groups. Serine donates a one-carbon group into the folate cycle through a reaction catalyzed by GlyA, which converts THF to 5,10-methylene-THF and generates glycine. In turn, methylene-THF can be converted to 5-methyl-THF or 10-formyl-THF to generate methyl- or formyl-group donors, respectively. Finally, 5-methyl-THF is used for the synthesis of methionine, and 10-formyl-THF provides the formyl group for the synthesis of purines and formylation of the amino acid attached to the initiator tRNA^fMet^ (although in mitochondria it is often denoted as tRNA^Met^, for simplicity, we refer to it as tRNA^fMet^) in bacteria and eukaryotic organelles. Together, the need for methionine and the formyl group form an important link between OCM and translation initiation.

Serine, which is the major source of the one-carbon units for OCM, is derived from glucose. Thus, the activity of OCM is directly linked to the availability of carbon and nitrogen sources. Further, the folate moieties (DHF and THF), which carry one-carbon units, possess polyglutamate (polyGlu) chains (linked through isopeptide bonds) of varying lengths of 1 to 11 residues (49, 50) which are important for their activity in OCM (51). Glutamate is a crucial amino acid in cells as it is required to fix ammonia (to produce glutamine), and it donates nitrogen for the synthesis of purines and pyrimidines as well as the amino groups to most amino acids. This dependence of OCM activity on glutamate levels enables OCM to act as a sensor of cellular biosynthetic capacity. As shown in Figure 2, many of the biochemical conversions in folate metabolism require redox regulators such as NAD^+^/NADH. Thus, besides the carbon and nitrogen sources, the activity of OCM is also regulated by the cellular redox status.

## Impact of the metabolites of one-carbon metabolism on translation

**Figure 3:**
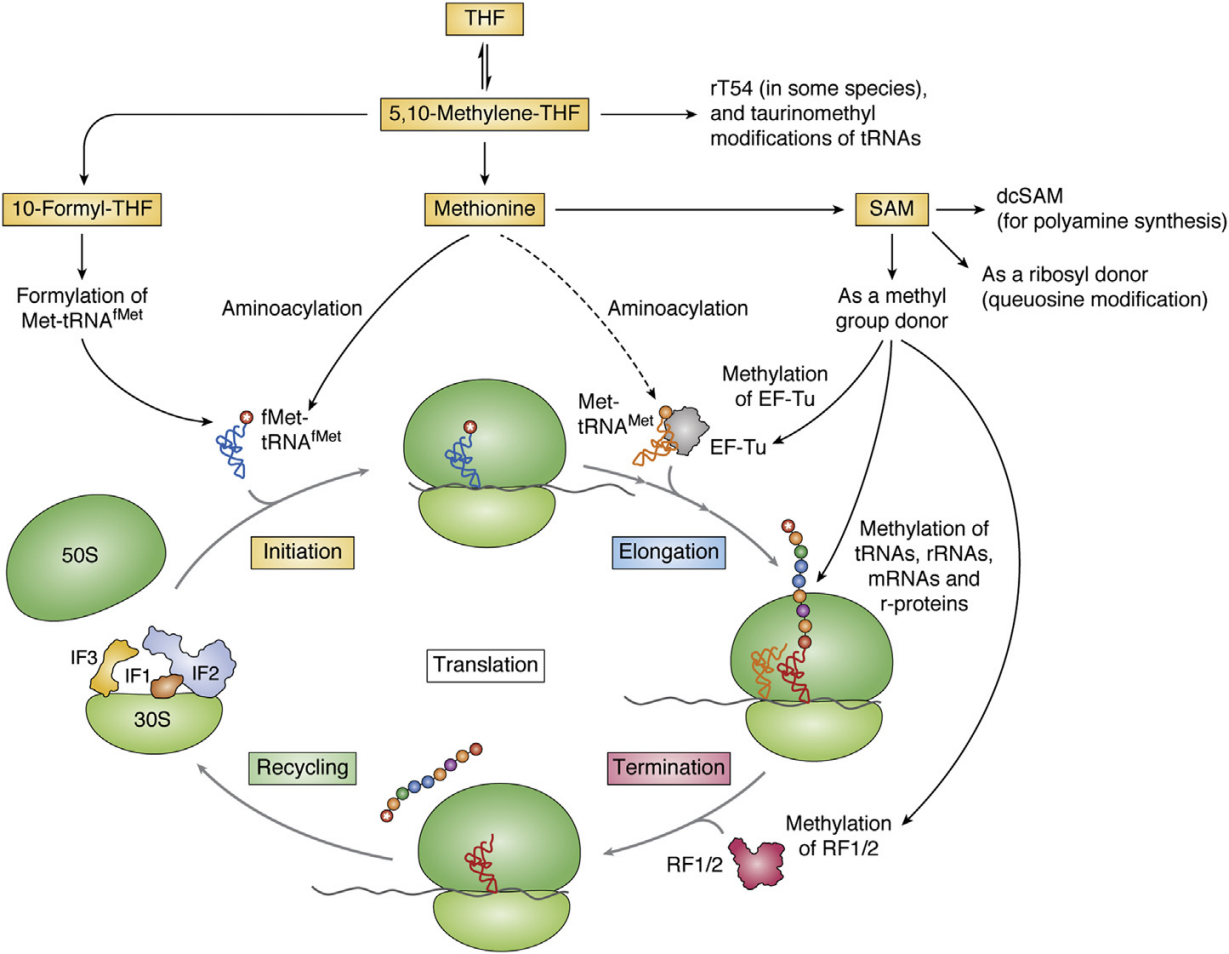
**Impact of one-carbon metabolism in translation:** The role of major products of the one-carbon metabolism in translation.

The metabolites in OCM (methionine, SAM, 5,10-methylene-THF, and 10-formyl-THF) affect various aspects of translation (Fig. 3). From recent studies, it is evident that OCM not only regulates the rate of protein synthesis but also affects the fidelity of translation. In turn, such a regulation by OCM intermediates might play indirect roles in the survival of cells under stressful conditions. In the following sections, we discuss how OCM metabolites modulate translation at various steps.

### Methionine and other amino acids

OCM regulates the synthesis of several amino acids, both directly and indirectly. It synthesizes glycine from serine, and methionine from homocysteine, which indirectly affects the levels of cysteine. The cellular levels of the amino acids may in turn affect the aminoacylation of the corresponding tRNAs (52, 53). More importantly, methionine is needed for aminoacylation of both the initiator tRNA^fMet^ and the elongator tRNA^Met^. Thus, it contributes to regulation at both the initiation and elongation steps. In all life forms, the N-terminal methionine is removed from most of the polypeptides by methionine aminopeptidases (MAP) (54). In bacteria, where the N-terminal methionine is formylated, MAP acts after the deformylation reaction (55). Given that the initiating methionine is removed from most of the proteins, the question of why methionine should be retained as the universal initiating amino acid remains a puzzle. Reporter assays in bacteria show that it is possible to initiate with amino acids other than methionine (56, 57, 58, 59), making the conservation of methionine as the initiating amino acid even more intriguing. One plausible explanation is that, in energetic terms, it is relatively more expensive to make methionine than the other amino acids, and therefore, it synchronizes the rate of translation initiation with the energy status of the cell (60). Also, as methionine has a thio-group, it may serve as a sensor of cellular sulfur contents. In addition, the synthesis of methionine requires homocysteine, which is also needed for cysteine synthesis. Thus, methionine levels indirectly regulate cysteine, which in turn affects various sulfur-containing modifications in tRNAs (61, 62). In yeast, deficiency of methionine leads to the deficiency of 2-thio modification at U34 residue of the lysine, glutamate, and glutamine tRNAs (62). While this deficiency leads to only a minor defect in translation, it results in altered carbon metabolism. Further, it has been observed that methionine supplementation under amino acid limited condition leads to transcriptional reprogramming to promote anabolic processes (63). Another major need for methionine is to synthesize the universal methyl donor, S-adenosylmethionine (SAM).

### S-adenosylmethionine (SAM)

SAM is the primary donor of methyl groups for various methyltransferases. Among different types of RNAs, tRNAs are the major targets of methylations (1, 64, 65). Methylations in tRNAs occur at different bases or the sugar moieties of the nucleosides (66) and play important roles in tRNA stability, recognition of cognate codon–anticodon pairs as well as their aminoacylation (67, 68, 69). Interestingly, SAM is also required for another modification, called queuosine, which occurs at G34 of GUN anticodon containing tRNAs (Asp, Asn, His, and Tyr). However, in this reaction, SAM is used for the transfer of its ribosyl moiety (70, 71). The queuosine modification protects tRNAs from RNases and stabilizes the codon–anticodon pairing to prevent frameshifting and read-through of codons (72, 73). In the case of mitochondrial tRNA^fMet^, SAM is required for a specialized modification at the wobble position, *i.e.*, 5-formylcytidine at C34. This modification is crucial for initiation from AUA codons in mitochondria by tRNA^fMet^ (74), a loss of which leads to translation defects and combined mitochondrial respiratory chain complex deficiency in humans (75, 76).

**Figure 4:**
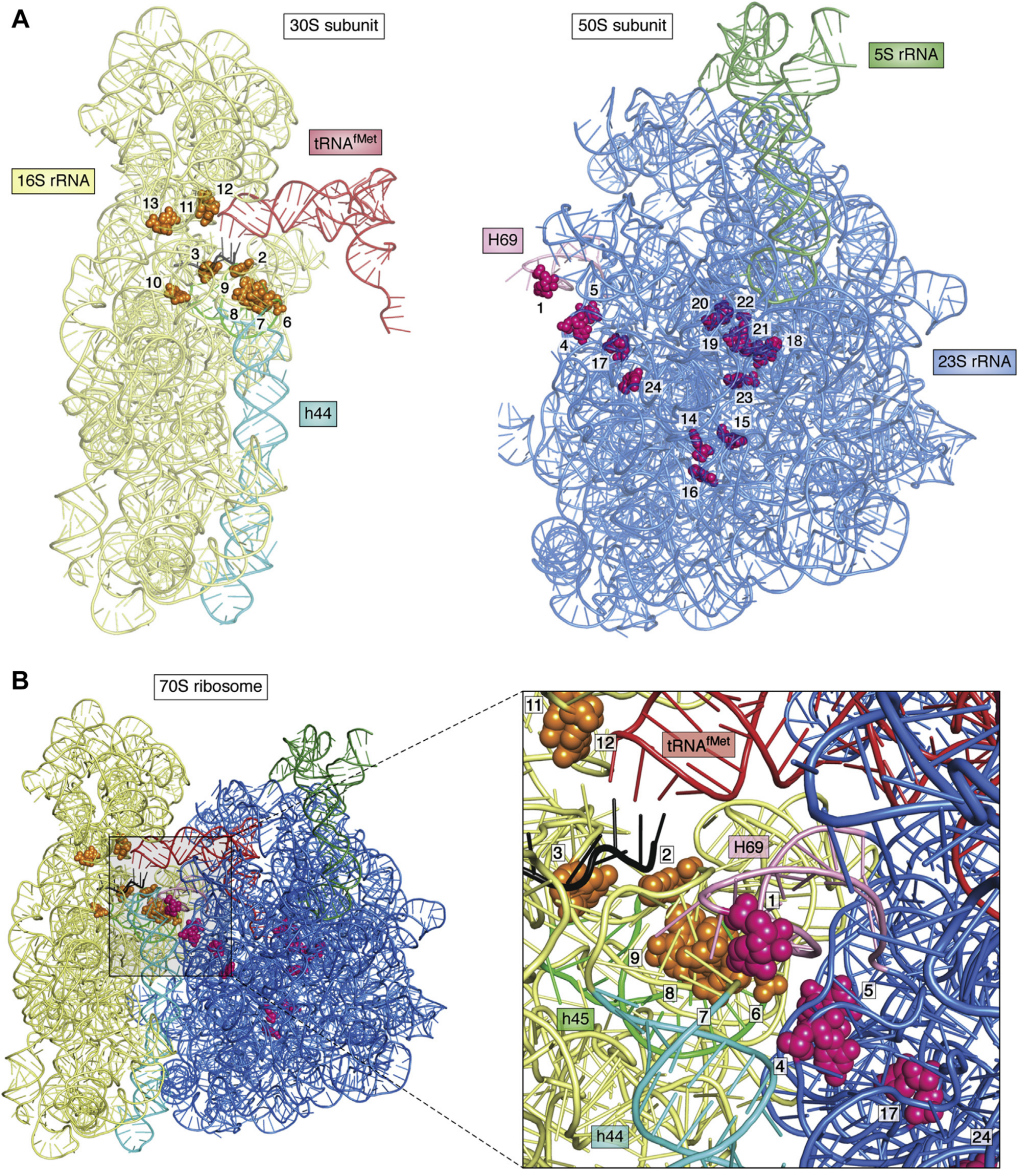
**The locations of rRNA modifications:** (*A*) The tertiary structures of 16S (in 30S) and 23S and 5S (in 50S) rRNA backbones within the 70S ribosome in *E. coli* (PDBID: 6GXM (183)) are shown using PyMOL. The methylated rRNA residues have been highlighted by the clusters of spheres and numbered. The methylated nucleosides in the 16S and 23S rRNAs are shown in orange and pink, respectively. The methylated nucleosides are numbered as follows: In 30S subunit: 2-m^3^U1498, 3-m^4^Cm1402, 6-m^2^G1516, 7-m^6^_2_A1518, 8-m^5^C1407, 9-m^6^_2_A1519, 10-m^7^G527, 11-m^2^G966, 12-m^5^C967, 13-m^2^G1207; In 50S subunit: 1-m^3^Ψ1915, 4-m^5^C1962, 5-m^2^G1835, 14-m^1^G745, 15-m^5^U747, 16-m^6^A1618, 17-m^5^U1939, 18-m^6^A2030, 19-m^7^G2069, 20-Gm2251, 21-m^2^G2445, 22-Cm2498, 23-m^2^A2503, 24-Um2552. In 30S, the P-site bound tRNA (*red*), the h44 (*turquoise*), and h45 (*pale green*); and in 50S, the 5S rRNA (*green*), H69 (*purple*), and 23S rRNA have been indicated. *B*, the 70S ribosome and the enlarged view focusing on the intersubunit region to show the location of methylated residues and the interaction between the h44, h45, and H69.

SAM is also required for methylation of various residues in rRNAs, which play important roles in ribosome biogenesis and function. The clustering of the methylations around the functionally important regions of ribosomes such as the intersubunit bridge regions, the decoding center, and the PTC (Fig. 4) suggests that they play important roles (77, 78, 79). In *E. coli,* 16S rRNA contains ten, and 23S rRNA contains 14 methylated residues, all of which require SAM as a methyl group donor (80). Most of these modifications are individually dispensable in *E. coli* (81). Although not essential for survival, many of these modifications affect the fidelity of translation initiation (discussed later) and elongation. For example, the m^2^G966 and m^5^C967 modifications in 16S rRNA enhance tRNA^fMet^ binding during initiation and prevent frameshifting during elongation (82, 83). The m^4^Cm1402 of 16S rRNA is present in the ribosomal P-site and might affect the interaction of the tRNA^fMet^ anticodon with the start codon (79). In 23S rRNA, the m^1^G745 is important for translation efficiency, and lack of the enzyme that methylates this residue (RlmA) reduces cellular growth rate (84).

Recently, methylations in mRNAs have emerged as major regulators of translation and stability of individual mRNAs. Although various types of methylations are found in eukaryotic mRNAs, only the presence of m^6^A has been reported in mRNAs from *E. coli* and other Gram-negative bacteria (85, 86). Interestingly, in *E. coli*, the m^6^A modifications are mainly clustered in energy metabolism-related mRNAs and small RNAs (85). Methylations also serve as posttranslational modifications in several of the translation associated proteins. The ribosomal proteins L3 (Q150), L7/L12 (K81), and L11 (K3, K39) are methylated using SAM (87). The roles of these methylations are not very clear, but they may be important in ribosome biogenesis. The 30S protein S12 is methylthiolated by RimO, a modification that is important for the A-site tRNA selection (88, 89, 90). In bacteria, elongation factor EF-Tu is methylated at position K56, which reduces GTP hydrolysis and may impact the translation accuracy under stress conditions (91). SAM is also required for methylation of catalytically crucial GGQ motif of release factors and promotes hydrolysis of the peptidyl-tRNA to release the polypeptide during the termination step (92, 93, 94). In addition, SAM is utilized in the synthesis of polyamines, which affect translation read-through and frameshifting (95, 96, 97, 98).

### 10-Formyl-THF

As folate transporters are absent in *E. coli*, direct uptake of 10-formyl-THF does not occur. It is therefore an essential metabolite generated by OCM. Besides its role in purine biosynthesis, 10-formyl-THF serves as the formyl group donor for formylation of Met-tRNA^fMet^ (by formylase, Fmt) in bacteria, mitochondria, and chloroplasts (Fig. 3). Formylation facilitates enhanced binding of fMet-tRNA^fMet^ to IF2 and its localization in the ribosomal P-site (99, 100). Formylation of Met-tRNA^fMet^ may be more crucial for initiation with 70S ribosomes as opposed to the canonical form of initiation that begins with 30S subunits (101, 102). The deletion of the Fmt gene in *E. coli* drastically reduces its growth (100, 103, 104, 105). However, formylation was found to be dispensable in other bacteria such as *Pseudomonas, Streptococci,* and *Bacillus* species (106, 107). At least in the case of *Pseudomonas aeruginosa*, it has been shown that it harbors a variant of IF2, which can support formylation-independent translation initiation (108). In *E. coli*, inhibition of OCM by trimethoprim (TMP) hinders protein synthesis *via* loss of formylation of Met-tRNA^fMet^ (5, 6). Analyses of mutants that grew on TMP-containing minimal media (supplemented with all the metabolites of OCM) revealed that the loss of methylation of U54 to ribothymidine (rT54) in tRNA^fMet^ facilitated initiation in the absence of formylation (6). However, another study observed that the absence of rT54 (because of the mutation in *trmA* gene, which methylates U54) could not rescue the function of the formylation defective tRNA^fMet^ mutants in reporter assays (109). Further investigations are needed to understand the possible role of U54 in formylation-independent initiation by Met-tRNA^fMet^.

The presence of the formyl group at the N-terminus of at least some of the newly synthesized proteins (e.g., microcin C peptides) could be important for their function (110). However, in most of the proteins, the N-terminal formyl group has an adverse effect on their functions and is efficiently removed soon after their translation, by deformylase (Def/PDF) (111). The importance of the Def function is highlighted from the occurrence of an antibiotic, actinonin, expressed from *Streptomycetes*, which targets Def (112). Mutations in FolD, GlyA, or Fmt can also lead to actinonin resistance (113, 114, 115). The deficiency of GlyA or FolD could reduce the cellular levels of 10-formyl-THF and thus formylation (9). Likewise, mutations in Fmt, which compromise its activity, also lead to gain of actinonin resistance. These observations suggest that the beneficial effects of formylation in enhancing translation initiation may be less critical than the need for its (formyl group) removal from the polypeptides (115). Consistent with this, expression of Fmt in the cytoplasm of yeast causes toxicity. However, the toxicity could be rescued by overexpression of Def (116). Recently, it has been shown that the N-terminal formyl group might act as a degradation signal and assist in degradation of mistranslated polypeptides (117). Interestingly, at least in yeast, in the stationary phase, mitochondrial FMT1 does formylate the aminoacylated cytosolic initiator tRNAs, and the translated proteins having N-terminal formylation are targeted for degradation (118, 119). In bacteria, activities of Fmt and Def could also be important in the recycling of the one-carbon unit (formate), and a perturbation of this recycling might affect the overall cellular metabolism. In *Staphylococcus aureus*, the deletion of the Fmt gene increased the NAD^+^/NADH ratio and reduced the consumption of glucose, arginine, and branched-chain amino acids indicating the importance of formylation in metabolic homeostasis (120).

The impact of the deficiency of formylation of Met-tRNA^fMet^ has also been investigated in the mitochondrial function. While, in yeast, the deletion of formylase (FMT1) had no severe phenotype (121, 122), in mouse fibroblasts, its (MTFMT) deletion decreased the synthesis and assembly of oxidative phosphorylation complexes (123). In Jurkat cells, perturbation of OCM by the deletion of SHMT2 (a GlyA orthologue) decreased both the mitochondrial respiration and translation by reducing formylation of mitochondrial Met-tRNA^fMet^ (11). However, another study noted that the deletion of SHMT2 or MTHFD2 (FolD homolog) in colon cancer cell line HCT116 (12) did not affect Met-tRNA^fMet^ formylation. Such different outcomes of compromising OCM may be due to genetic differences in the cell lines or their metabolic adaptations. The presence of the formyl group at the N-terminal of a mitochondrial protein COX1 contributes to the assembly of the cytochrome C complex (124). In humans, mutations in formylase lead to Leigh syndrome, a neurometabolic syndrome (125, 126, 127). Thus, the impact of the formylation of mitochondrial Met-tRNA^fMet^ might be tissue-specific, detailed analyses of which is still lacking.

### 5,10-Methylene-THF

5,10-methylene-THF is a key intermediate for methionine, thymidylate, and 10-formyl-THF production. The 5,10-methylene-THF is also used as methyl donor for tRNA modifications such as taurinomethylation in mitochondria and some bacteria (128). In mitochondria, U at the wobble position of Leu, Lys, Glu, Gln, and Trp tRNAs gets 5-taurinomethyluridine modification while a subset of these (Lys, Glu, and Gln tRNAs) gets 5-taurinomethyl-2-thiouridine modification. Deficiency of the taurinomethyluridine modification leads to stalling of mitochondrial ribosomes on AAG and UUG codons (12). The lack of taurinomethyluridine modifications has also been reported in the mutant tRNAs responsible for mitochondrial encephalopathies of MERRF (myoclonus epilepsy associated with ragged-red fibers) and MELAS (mitochondrial myopathy, encephalopathy, lactic acidosis, and stroke-like episodes) (129, 130, 131). The 5,10-methylene-THF is also used for rT modification of U54 residue of tRNAs in some species of *Bacillus* and *Streptococci* (132).

## One-carbon metabolism and regulation of the fidelity of translation initiation

OCM intermediates regulate/fine-tune mRNA translation in multiple ways (Fig. 3) impacting various steps (initiation, elongation, termination, and ribosome recycling) in protein synthesis. However, translation initiation is considered as the rate-limiting step and is tightly regulated with respect to changes in the environment/nutrient availability. Translation initiation uses a specialized tRNA, the initiator tRNA. In the following section, we briefly discuss the structure–function aspects of this tRNA to better understand the regulation of initiation by OCM.

### Initiator tRNA^fMet^

**Figure 5:**
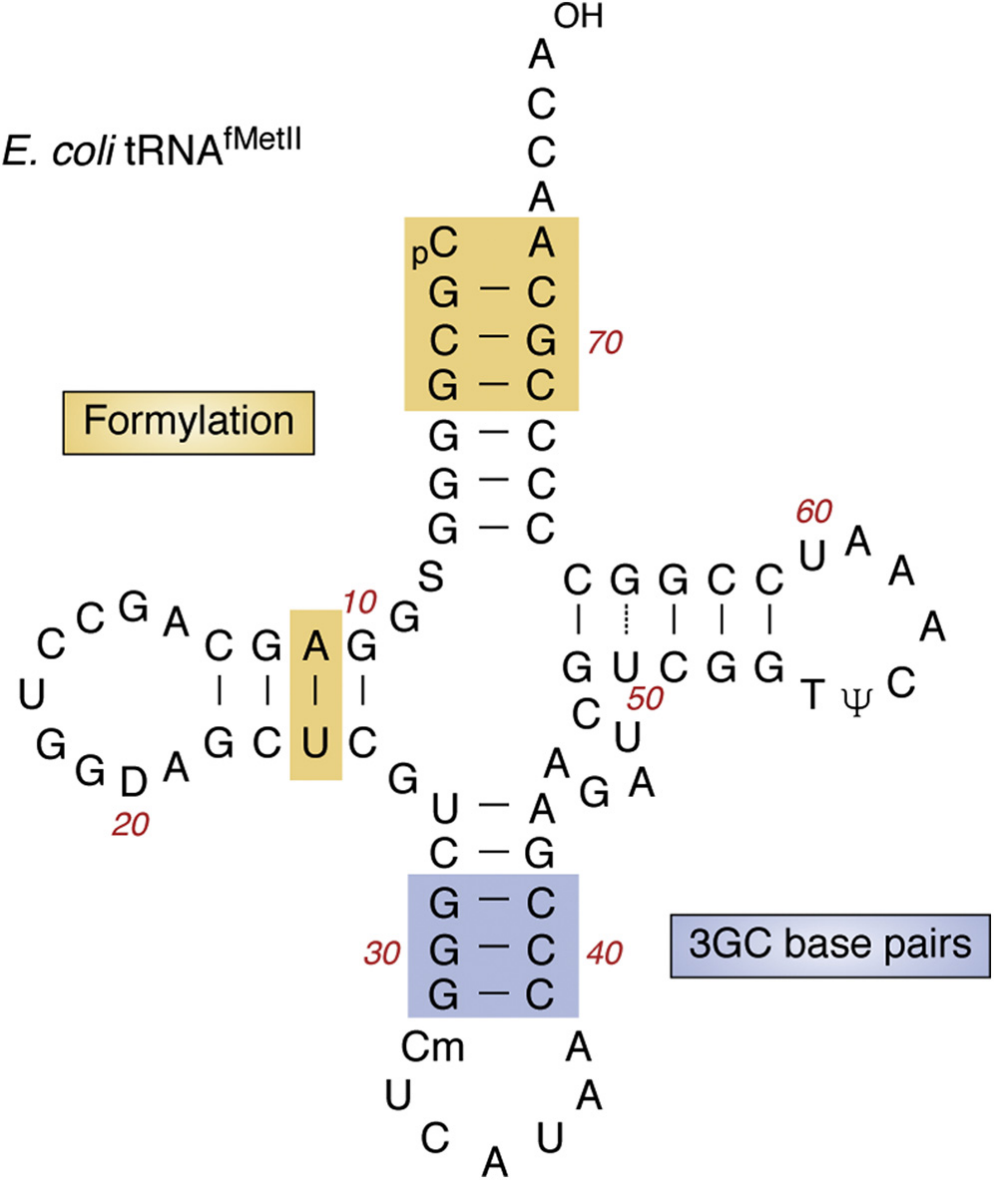
**Unique features of tRNA**^**fMet**^**.** The cloverleaf structure of tRNA^fMetII^ (from *metY* in *E. coli* K strains) is shown. The tRNA^fMetI^ in *E. coli* possesses methylated G at position 46 instead of A. The features important for the formylation of the methionine attached to tRNA^fMet^ are shown in *brown*. The three GC base pairs are highlighted in *blue*.

In *E. coli*, the initiator tRNAs are encoded by four genes. Three of these (*metZ*, *metW*, and *metV*) encoding tRNA^fMetI^ occur at 63.5 min location, and the fourth (*metY*) encoding tRNA^fMetII^ occurs at 71.5 min location in the genome. The initiator tRNAs differ from elongator tRNAs in their ability to directly bind the ribosomal P-site during initiation. Initiator tRNA and the accuracy of its selection in the ribosomal P-site are crucial for the efficiency of translation initiation and to determine the correct reading frame in an mRNA (133, 134). Initiator tRNAs in bacteria (tRNA^fMet^) have two major structural features that facilitate its binding in the P-site (Fig. 5). The first one is the presence of a mismatched base pair at the top of the acceptor stem (C_1_ x A_72_ in *E. coli*). The second one is the presence of the three consecutive GC base pairs (G_29_:C_41_; G_30_:C_40_; G_31_:C_39_, called 3GC base pairs) in the anticodon stem (135, 136, 137). One of the functions the mismatch in the acceptor stem of tRNA^fMet^ contributes to is in the formylation of the amino acid attached to it. The efficiency of the formylation of Met-tRNA^fMet^ is also assisted by the G_2_:C_71_, C_3_:G_70_, and A_11_:U_24_ base pairs. Unlike formylation (found in bacteria and eukaryotic organelles), the presence of the 3GC base pairs is highly conserved in initiator tRNAs in all domains of life, indicating its high-functional relevance (100, 134). Transplanting these two features (the C_1_ x A_72_ mismatch at the top of the acceptor stem and the 3GC base pairs in the anticodon stem) into elongator tRNAs, tRNA^Met^ and tRNA^Gln^, allows them to initiate translation in *E. coli* (138, 139). Genetic analyses have shown that these two features contribute to specific roles in initiation (100, 140). While the major function of formylation is to facilitate the initial binding of fMet-tRNA^fMet^ to the ribosome, the 3GC base pairs are crucial in transiting the tRNA from 30S IC to 70S IC (Fig. 1) (141).

### Role of the rate of formylation of Met-tRNA^fMet^

Formylation of Met-tRNA^fMet^ enhances its affinity to IF2, which allows preferential binding of tRNA^fMet^ in the P-site (99, 142). To understand the importance of formylation, we have used a mutant tRNA^fMet^ called the “3GC mutant” wherein the highly conserved 3GC base pairs in the anticodon stem were substituted by the sequence found in the elongator tRNA^Met^ as a model to investigate the fidelity of tRNA selection at the step of initiation (143). Using this model, we observed that an *E. coli* mutant expressing low levels of Fmt (consequently a low rate of formylation) permitted initiation with the 3GC mutant tRNA (105). As Fmt expression was increased, participation of the 3GC mutant tRNA in initiation was diminished. A simple explanation for this observation is that the lack of formylation decreases the affinity of the aminoacylated tRNA^fMet^ to the P-site (because of its poor binding to IF2), leading to the vacant P-sites in the available ribosomes. Under such conditions, other tRNAs (including the 3GC mutant tRNA^fMet^) may now begin to occupy the P-sites in the available ribosomes (not already bound with the initiator tRNA) and initiate protein synthesis. Such an interpretation is consistent with the observation that the deletion of the three initiator tRNA genes (*metZWV*) allows initiation with not only the 3GC mutant tRNA^fMet^ but also elongator tRNAs (tRNA^Gln^, tRNA^Pro^, tRNA^Glu^) (134, 144). Interestingly, under the Fmt deficient conditions, the tRNA^fMet^, despite possessing the C_1_ x A_72_, participates at the step of elongation (through binding to EF-Tu) (105, 145). Thus, the rate of formylation of the aminoacylated tRNA^fMet^ is crucial in determining whether it would function strictly in initiation or it could also participate at the step of elongation (145). The rate of formylation can be controlled by the availability of either Fmt (105) or 10-formyl-THF (9). Interestingly, in the human mitochondria where a single tRNA^Met^ serves as both initiator and elongator, a wobble pair (G_1_-U_72_) at the 1-72 position results in its partial formylation (138, 146), allowing the aminoacylated tRNA to bind to either IF2 (in the formylated form) or EF-Tu (in unformylated form) for its participation in initiation and elongation steps, respectively. In the case of mitochondria from *Trypanosoma brucei*, cytosolic elongator tRNA^Met^ is used both in initiation and in elongation (147). Although the cytosolic tRNA^Met^ in *T. brucei* possesses a G-C base pair at the 1-72 position, it acts as an initiator tRNA when partially formylated. Likewise, *E. coli* strains deleted for all four of its initiator tRNA genes and both of the elongator tRNA^Met^ genes could be obtained in the presence of initiator tRNA mutants compromised for formylation (148). In these *E. coli* strains, as in mitochondria, a single tRNA served the functions of both the initiator and elongator tRNA^Met^. Thus, the rate of formylation can determine the fate of the tRNA^fMet^ in its participation at the steps of initiation and elongation.

In *E. coli*, the deletion of the *fmt* gene leads to a drastic decrease in growth rate, most likely because of the low rate of initiation and undesired initiation with the elongator tRNAs (100, 103). Interestingly, the compromised growth rate is rescued by overexpression of tRNA^fMet^ (100, 115, 145), suggesting that the occurrence of multiple initiator tRNA genes in most of the organisms not only enhances the rate of initiation but also decreases chances of initiation with the elongator tRNAs. Studies have shown that a high level of initiator tRNA is indeed needed to outcompete elongator tRNA binding in the P-site (134, 144). Thus, both formylation and abundance of tRNA^fMet^ regulate fidelity of translation initiation.

### Role of methylations in rRNA in initiation

The role of rRNA methylations in the fidelity of translation initiation was initially indicated by the mutations in the OCM enzyme, FolD, in *E. coli.* FolD is involved in the synthesis of 10-formyl-THF from 5,10-methylene-THF. A mutation in *folD* gene (G122D mutation in FolD) allowed initiation with the 3GC mutant tRNA^fMet^, which is an indication of the loss of fidelity at the step of initiation (149). Detailed analyses of this strain suggested that the lack of methylations in rRNA (besides the lack of formylation of tRNA^fMet^, discussed above) also contributed to initiation with the mutant tRNA^fMet^. In fact, the deletion of methyltransferases specific for various positions in 16S rRNA such as RsmC (m^2^G at 1207, located in the A-site), RsmE (m^3^U at 1498, present in the P-site), and RsmF (m^5^C at 1407, located in the vicinity of mRNA binding region) in *E. coli* allowed initiation with the 3GC mutant tRNA^fMet^. However, the mechanism of cross talk between the rRNA methylations and the 3GC base pairs of the initiator tRNA has remained unclear.

Among the 16S rRNA residues that are methylated, G966 and C967 are in the direct vicinity of tRNA^fMet^ in the P-site. The G966 and C967 are methylated by RsmD and RsmB, respectively. The methylated G966 residue is found to cross-link with the position C34 of tRNA^fMet^ in the P-site (150). In the structure of the 70S ribosome, G966 and C967 form a stacking interaction with the C34 of the tRNA^fMet^ (37, 81). *In vitro* studies suggested that the lack of these two methylations affects the initiation complex formation and reduces the binding of tRNA^fMet^ by twofold (83). Further, these methylations play an important role in the binding of elongator tRNAs *in vitro*. *In vivo*, the absence of RsmB or RsmD allows increased initiation with the 3GC mutant tRNA^fMet^ (151). The strain deleted for RsmB/D also shows cold sensitivity and ribosome biogenesis defect, which may also cause some leakiness in the fidelity of initiation and relaxed scrutiny of P-site bound tRNA (151).

Another important methyltransferase that contributes to initiator tRNA selection in the P-site is RsmA (KsgA). RsmA modifies residues A1518 and A1519 of 16S rRNA. The absence of these methylations (m^6^A1518 and m^6^A1519 in the 3’ terminal helix of 16S rRNA) allowed enhanced initiation with the 3GC mutant tRNA^fMet^ (152). These modifications are also important for the 30S ribosome biogenesis as well as subunit interactions (152, 153). The rate of the subunit interaction also influences the fidelity of the initiator tRNA selection by affecting the dissociation rate of initiation complex (154). *In vitro*, the increased rate of the 50S joining to 30S IC could stabilize elongator tRNAs in the ribosomal P-site (154, 155). The subunit interaction is also influenced by factors such as IF1, IF3, and RRF, which dissociate 70S ribosomes (156). *In vivo*, compromising RRF activity decreases the fidelity of initiation (157). Combining perturbation of OCM (by *folD122* mutation) or loss of methylations at A1518/A1519 (by deletion of *rsmA*) with compromised RRF activity (RRF^ts^) severely reduced the fidelity of initiation (157). Thus, the RRF and methylations in the intersubunit region play an important role in maintaining the fidelity of translation initiation. Another mechanism by which RsmA could regulate fidelity of initiation is *via* IF3. The methylated 1518/1519 region also serves as the binding site for IF3. As IF3 is known to be involved in the selection of the 3GC base pairs and dissociation of the ribosomal subunits, the compromised IF3 binding leads to loss of fidelity of initiation (158). The genetic interactions between IF3, RRF, and FolD support that the OCM affects the fidelity of translation initiation by multiple mechanisms (157).

Other than their roles on tRNA^fMet^ selection in the ribosomal P-site, the rRNA methylations also affect the ribosome biogenesis, which in turn affects the fidelity of translation initiation (159). The perturbation of SAM synthesis, by deletion of *S*-ribosylhomocysteine nucleosidase (Mtn) gene, affected the 50S biogenesis *via* 2′-O-methylation at position 2552 (Um2552) of 23S rRNA by RlmE (160, 161). In *E. coli*, cold temperature leads to ribosome biogenesis defect and accumulation of immature 30S subunits with unprocessed ends. The cold temperature also increases the initiation with the 3GC mutant tRNA^fMet^, thus compromising the fidelity of initiation (140). We also found that 3GC mutant tRNA^fMet^ can lead to the 30S biogenesis defect at the cold temperatures (162). Further, overexpression of 3GC mutant tRNA^fMet^ enhanced the ribosome biogenesis defect in the absence of RsmB/D deletion or deletion of S9 C-terminal tail. These results suggest that fidelity of translation initiation is also dependent on ribosome biogenesis (163).

### Role of methylations in the initiator tRNA

The function of tRNA^fMet^ *per se* is also linked to OCM through several modifications. As discussed, rT54 modification in the tRNAs is dependent on either SAM or 5,10-methylene-THF in different bacteria. Whether this modification assists in the fidelity of tRNA^fMet^ selection under varying formylation conditions needs further investigation. In *E. coli* K-strains, the tRNA^fMetI^, which contributes 75% of the total tRNA^fMet^ population, possesses methylated G at position 46 in the variable loop while the minor form tRNA^fMetII^ possesses A46 at this position. In *E. coli*, the m^7^G modification at position 46 is performed by YggH using SAM (164). Further, the anticodon loop of tRNA^fMet^ possesses a SAM-dependent 2′-O-methylation of cytidine (Cm) at position 32 carried out by YfhQ (165). This methylation might play a role in stabilization of the anticodon loop of the tRNA^fMet^.

## The physiological relevance of the regulation of translation by one-carbon metabolism

As discussed, OCM affects the rate and fidelity of translation *via* the translational apparatus in multiple ways (*e.g.*, availability of amino acids, modifications of translation factors, tRNA, rRNA, ribosomal proteins, and mRNAs, Fig. 3). The physiological relevance of these regulations may be in dealing with various types of stress, which a cell needs to adapt to and cope with the altered environment. For example, cells maintain the translation of a subset of mRNAs that are required in the specific conditions while inhibiting the bulk translation or synthesize novel forms of proteins by altered initiation to survive under stress conditions. A perturbation in nutrients or redox status of the cell can perturb OCM flux, which in turn may lead to the deficiencies in the cofactors such as 10-formyl-THF, 5,10-methylene-THF, or SAM. The consequence of this could be that only a fraction of the population of the molecules in the translational apparatus is modified. Also, as there are multiple sites of modifications particularly in the rRNAs and tRNAs, modification of a fraction of the population of the molecules at the nonoverlapping positions can lead to generation of a remarkable heterogeneity in the translation apparatus. Such heterogeneity may lead to preferential translation of select mRNAs or even a loss in the fidelity of translation (166). The heterogeneity in the ribosomes may arise not only from the modifications at various positions in its RNAs but also in the form of the composition of the ribosomal proteins or rRNA sequence/length under various growth/physiological conditions (166, 167, 168). Both the phenomenon of preferential translation of a subset of mRNAs and leakiness in the fidelity of translational reading frames could impact the fitness of the organisms without having to have any changes in their genomes (169).

The presence of various mechanisms for modulation of the fidelity of translation initiation by OCM might represent evolutionary strategies to reprogram gene expression to remain in synchrony with the cellular metabolism. A slight loss of fidelity might cause mistranslation by altering the start codon recognition or internal codon selection. Such altered translation provides a phenotypically variable but genotypically stable population to cope with the stress conditions (170, 171, 172, 173, 174, 175, 176). An increase in general mistranslation has been shown to be advantageous under DNA damaging conditions by faster activation of the SOS response (175). An *E. coli* strain possessing slightly lower level of tRNA^fMet^ had a competitive advantage under low nutrition conditions (177). In *Salmonella*, the expression of VapC, which cleaves tRNA^fMet^ and promotes initiation from elongation codons, is important for its virulence (178). These observations suggest that under stress conditions, bacteria may employ alternate initiation strategies to translate stress response genes or increase proteome diversity (169). High accuracy of translation found in certain high-fidelity translational elongation mutant strains (restrictive strains) of *E. coli* reduces growth (179). Thus, having tunable factor functions provides an advantage for cells to cope with the stress conditions. One way to generate proteome diversity is to use alternative start codons to read alternative ORFs within the same mRNA. Such alternative initiation mechanisms are shown to play an important role in cancer cells (180, 181).

## Perspective

Cells have evolved various mechanisms to tightly coordinate translation with cellular metabolism. The regulation of translation by OCM, an ancient pathway, is a conserved phenomenon from bacteria to humans. The coupling of OCM and translation might have existed even in very early forms of life/LUCA (last universal common ancestor). The presence of a polyglutamyl chain (49, 50) in the folates might be a sign of preribosomal form of protein synthesis and may well be among the earliest forms of protein synthesis linking it to OCM. Furthermore, as glutamate can be generated from α-ketoglutarate (a metabolite in the tricarboxylic acid (TCA) cycle), the dependence of polyglutamyl chain in the folate derivatives also extends the impact of OCM to the carbohydrate metabolism (51). The cross talk between the TCA cycle and OCM may be very interesting in the context of mitochondrial translation as the TCA cycle is perturbed in various cancers (182). However, detailed investigation on the role of OCM on the fidelity of translation in mitochondria is not yet feasible due to the lack of *in vivo* reporter systems. The modulation of the OCM activity with various environmental conditions and the corresponding modifications in the translation apparatus (including heterogeneous ribosomes) need more investigations. It would be interesting to better understand the implications of how ribosomal heterogeneity (rRNA modifications) or the specialized ribosomes offer physiologically relevant solutions to cellular needs especially under changing nutrient conditions. Many of these conditions may lead to initiation with elongator tRNAs resulting in the production of novel/unusual peptides. Finally, we believe that the detailed systems biological, biochemical, and molecular genetics studies will not only result in a better understanding of the cross talk between the two ancient pathways of protein synthesis and OCM but also provide novel strategies (such as synthetic combinations) to overcome antimicrobial drug resistance mechanisms.

## Conflicts of interest

The authors declare that they have no conflicts of interest with the contents of this article.
